# The Reality in the Surveillance of Breast Cancer Survivors—Results of a Patient Survey

**DOI:** 10.4137/bcbcr.s511

**Published:** 2008-02-26

**Authors:** Stemmler Hans-Joachim, Lässig Dorit, Stieber Petra, Bauerfeind Ingo, Kahlert Steffen, Fasching Peter Alexander, Beckmann Matthias Wilhelm, Glattes Margrit, Goldmann-Posch Ursula, Hoffmann Verena, Untch Michael, Heinemann Volker

**Affiliations:** 1 Department of Hematology and Oncology; 2 Department of clinical Chemistry; 3 Department of Gynaecology and Obstetrics; 4 Department of Gynaecology and Obstetrics, University of Erlangen, Germany; 5 AOK Rheinland/Hamburg, Germany; 6 Mamazone e.V., Augsburg, Germany; 7 Department of Gynaecology and Obstetrics, Helios Klinikum, Berlin-Buch, Germany; 1–3 Ludwig-Maximilians University of Munich, Klinikum Großhadern, Munich, Germany

**Keywords:** breast cancer, follow-up, guidelines, surveillance

## Abstract

**Background:**

International guidelines for the surveillance of breast cancer patients recommend a minimized clinical follow-up including routine history and physical examination and regularly scheduled mammograms. However, the abandonment of scheduled follow-up examinations in breast cancer survivors remains a contradiction to established follow-up guidelines for other solid tumours.

**Patients and Methods:**

We report the patients’ view on the basis of a survey performed in two separate geographical areas in Germany. The questionnaires were sent out to 2.658 patients with a history of breast cancer.

**Results:**

A total of 801 patients (30.1%) responded to the questionnaire. The results of the survey can be summarized in two major categories: First, necessity for surveillance was affirmed by a majority (>95%), and 47.8% of the organized patients answered that there was a need for more intensive diagnostic effort during follow-up. The main expectation from an intensified follow-up was the increased feeling of security as expressed by >80% of the women. Second, the present survey indicates that most of the regularly scheduled follow-up visits were expanded using extensive laboratory and imaging procedures exceeding the quantity of examinations recommended in the present follow-up guidelines.

**Conclusion:**

Despite the fact that only one third of the patients responded to the questionnaire, the survey indicates that a majority of physicians who treated these patients still do not accept the present follow-up guidelines. To some extent this may be explained by the observation that patients and possibly also their doctors trust that intensified follow-up increases diagnostic security and survival. Since considerable changes in the treatment options of breast cancer have been made during the last decades a new trial of investigations in follow-up is warranted.

## Introduction

The care of patients who survived their breast cancer requires an understanding of patterns of relapse as well as the unique medical and psychological needs that arise in this group of patients. One primary goal of surveillance is the early recognition and treatment of potentially curable disease recurrence. A majority of breast cancer recurrences occur during the first decade after primary therapy, particularly during years 2 to 5; however, they can occur much later [1–4]. A recent meta-analyses of 12 studies involving 5.045 patients found that 40% of patients with loco-regional recurrences were diagnosed during routine clinic visits, whereas the remainder (60%) developed symptomatic recurrences between the intervals of regularly scheduled clinical visits [5]. Recommendations regarding surveillance for disease relapse are based upon guidelines issued in an update 2006 from the American Society of Clinical Oncology (ASCO) [6]. These guidelines included routine history and physical examination and regularly scheduled mammograms and excluded intensified laboratory and imaging procedures for asymptomatic patients [6].

It has often been discussed if an intensified follow-up, expanded by intensive laboratory and imaging procedures improves the outcome of breast cancer patients and survivors. Two randomized trials and a Cochrance review have concluded that there is no survival benefit from more intensive surveillance during post-treatment follow-up [7–10]. Moreover, no study exists, which has evaluated the benefit of more frequent clinical visits in patients with known high-risk versus low-risk disease [6]. Finally, a follow-up that was not guideline-compliant costs 2.2 to 3.6 times more than a guideline-compliant follow-up [11, 12].

However, the abandonment of scheduled follow-up examinations in breast cancer patients and survivors remains a contradiction to established follow-up guidelines for other solid tumours. Taking into consideration that treatment options for breast cancer have improved continuously over the last years, it is mandatory to initiate a new surveillance study which investigates the influence of these improved possibilities on survival.

The present survey was performed to analyze the reality in the existential orientation of women with breast cancer in the phase of follow-up and to evaluate the patients’ view on the issue of surveillance after breast cancer in preparation for a new surveillance study.

## Patients and Methods

### Patient recruitment

The present analysis based on a patient survey initiated in two different states (Nordrhein-Westfalen and Bavaria) of Germany in the year 2005. A questionnaire was sent out to 2.658 patients with a history of breast cancer.

The selection of patients based on the databases of a self-help organization (Mamazone e.V., Augsburg, Germany) and a local health insurance company (AOK Rheinland/Hamburg, Germany).

Due to the completely anonymous questionnaire an approval by an ethics committee was not mandatory. There was no designated recall for non-responders. Among 801 patients who responded to the questionnaire, 476 (59.4%) were organized in a self-help group.

### The questionnaire

The questionnaire was developed in a cooperative effort of medical oncologists, gynaecologists and the German breast-cancer advocacy-group mamazone. The questionnaire included 29 questions with 58 variables and is accessible on the homepage of our department (http://med3.klinikum.uni-muenchen.de/ycms/Studien_80.htm).

### Statistical methods

The data of the survey were analyzed with descriptive statistical methods. Differences between patient groups were analyzed using the χ^2^-test (Chi-square test).

## Results

### Baseline characteristics

From 2.658 patients who were included in the survey a total of 801 patients replied to the questionnaire (30.1%). The median age amounts to 62 years (range 23–85 years). More than half of the respondents were members of a self-help organization (59.4%).

The majority of the patients were disease-free at the time of the survey (71.7%). Loco-regional recurrence was diagnosed in 13.4% and the remaining 14.9% had metastatic disease at the time of the survey.

### Follow-up: The patients’ view

**Figure 1:**
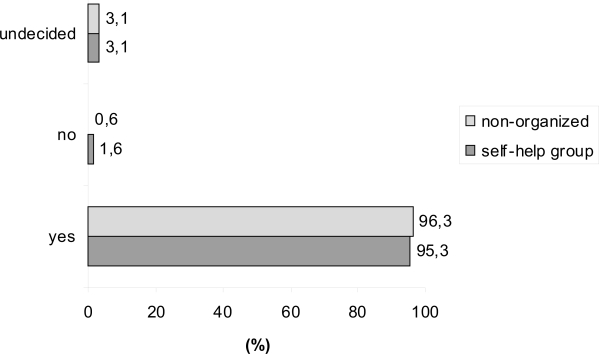
Question: Necessity of surveillance?

Independent of a membership to a self-help organization a majority of the patients affirmed the need for follow-up after primary treatment of breast cancer. Necessity of surveillance was affirmed by 95.3% of the members of a self-help group which was comparable to 96.3% in non-organized patients (Fig. 1).

**Figure 2:**
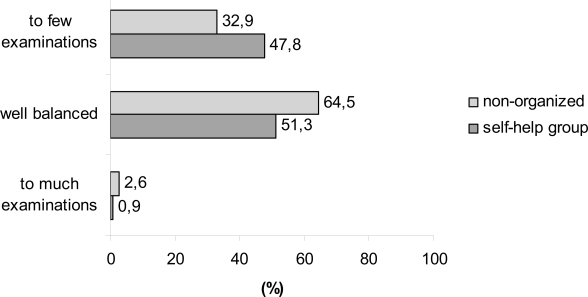
Question: Adequacy of the present surveillance regimen?

When asked for the adequacy of the present follow-up, nearly one-half of the organized patients (47.8%) and one-third of the non-organized patients (32.9%) asked for more laboratory and imaging procedures during their follow-up exams. About two third of the non-organized and the half of the organized patients accepted the present intensity of follow up (self-help group vs non-organized patients, 51.3 vs 64.5%; Fig. 2).

**Figure 3:**
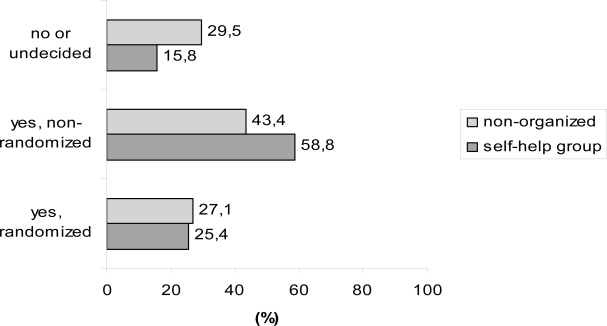
Question: Willing to participate in a surveillance study?

In view of the unsatisfactory evidence of clinical studies on follow-up in breast cancer the present survey includes the question if the afflicted women are willing to participate in a clinical study on follow-up. Only about a quarter of the patients (self-help group vs non-organized patients, 25.4 vs 27.1%) indicated that they would participate in a trial randomising between conventional and more intensive follow-up, whereas the majority would prefer participation in a single-arm, non-randomized trial including an intensified follow-up (self-help group vs non-organized patients, 58.8 vs 43.4%) (Fig. 3).

Independent of the membership to a self-help organization, more than 80% of the responders stated that regularly scheduled visits during follow-up are important to come to terms with the disease.

**Figure 4:**
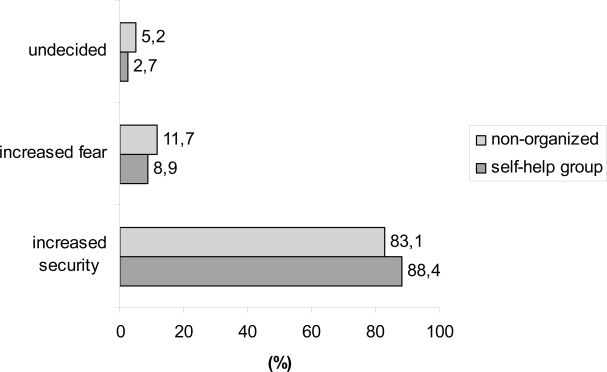
Question: Intensification of surveillance leads to…?

When asked for the consequences following from a more intensified follow-up, more than 80% of the patients stated that intensification of follow-up was associated with an increased sense of security (Fig. 4). And independent of the membership to a self-help organization, a majority of the patients in the surveys believed that earlier detection of any recurrence or metastatic spread will improve survival (97%, e.g. 95.8% of the organized patients).

When asked for the attitude to life a quarter of the organized (25%) and a third of the non-organized (33%) patients responded that they would prefer to neglect rather than to constantly analyze the disease. Moreover, about a third of the respondents stated that they feel confident and comfortable and the disease has been overcome (34%, e.g. 28% of the organized patients).

### Disease status and patients’ view

283 patients (35.3%) experienced loco-regional recurrence or metastatic disease during follow-up. Necessity for surveillance was affirmed by 98.3% of the responders. The overwhelming majority (90%) of these patients required more laboratory and imaging procedures during their follow-up exams.

### Follow-up—reality

When asked for the regularly scheduled follow-up visits in the past, it was obvious, that most of those visits were expanded with extensive laboratory tests and imaging procedures. Those procedures were offered significantly more often to patients who were organized members of a self-help group. A significant difference was determined for routine history, laboratory testing including tumor marker tests, and imaging procedures except CT or PET-CT scans, which were all performed more frequently in patients who were organized members of a self-help group (Table 1). It is noteworthy that the questionnaire asked for tests routinely offered to the patients regardless to the presence of clinical symptoms of loco regional recurrent or metastatic disease.

## Discussion

Nearly all national and international guidelines for follow-up of breast cancer patients do explicitly not recommend regular laboratory and imaging screening procedures for asymptomatic patients. According to these guidelines, follow-up should be focussed on the breast. Only patients with suspected tumour-related symptoms should be screened for metastatic spread. The American Society of Clinical Oncology 2006 update of the breast cancer follow-up and management guidelines in the adjuvant setting concluded, that careful history, physical examination and regular mammography performed by an experienced physician are appropriate for detection of breast cancer recurrence in asymptomatic patients [6].

Although an intensified follow-up may detect asymptomatic disease recurrence, two randomized trials and a Cochrane analysis failed to demonstrate a survival benefit from more intensive surveillance during post-treatment follow-up [7–10]. However, diagnostic tools and treatment options have improved continuously over the last years. Despite the fact that both trials in the 80’s were powered to draw the conclusions they made, some minor but also major concerns remained:

### Minor concerns

- Both studies are of limited value for patients with a higher risk of relapse since more than 50% of the included patients were node-negative and only a minority (<10%) of the patients had larger tumours (pT3 and pT4) [9, 10].
- Only one trial included regularly scheduled ultrasound studies of the liver at yearly intervals! Laboratory tests did not include tumour marker analyses [10].
- It is critical to consider that in both studies the relapse rate amounts to 20% at 5 years which is clearly in contrast to a higher relapse rate (relapse rate at 5 years up to 40%) as reported in other studies [13].

### Major concerns

- Considerable changes in surgical procedures, innovations in interventional radiology and an improvement in the systemic treatment options of breast cancer have been made in the last decades, which were all not considered in those trials [14–17]. For example, it has been demonstrated that an aggressive surgical approach improves the outcome of highly selected patients with oligometastatic breast cancer [17].

This non-representative investigation was initiated to evaluate two major issues: First to reflect the reality of follow-up as performed typically in women with a history of breast cancer and second, to evaluate the patients’ view. The results of this can be summarized in two major categories:

As demonstrated in Table 1, a majority of the physicians who treated these patients obviously does not accept the present follow-up guidelines. To some extent this may be explained by the observation that patients and possibly also their doctors trust that intensified follow-up increases diagnostic security. Unsurprisingly, those extensive laboratory tests and imaging procedures were offered significantly more often to patients who were organized members of a self-help group. This is partly explained by better informed patients who insist upon an intensified follow-up (Table 1).

Independent of the membership to a self-help organization, the overwhelming majority of the patients who responded to the questionnaire affirmed the general necessity for follow-up (>95%) and 47.8% of the organized patients answered that there was a need for more intensive diagnostic effort with regard to laboratory exams and imaging procedures during follow-up (32.9% of non-organized patients). Unsurprisingly, this percentage was much higher in patients with recurrent or metastatic disease (90%). Contrary to common belief more than 80% of the respondents stated that this intensification of follow-up was not associated with an increased sense of uncertainty but rather an increased sense of security. This has been consistently found in a Dutch study of de Bock et al. [18]. Nevertheless, it is critical to discuss that these data are in contrast to those of Gulliford et al. [19]. In their study patients were randomly assigned to a conventional schedule of clinic visits or to a reduced schedule of clinic visits only after mammography. Twice as many patients in both groups expressed a preference for reducing rather than increasing follow up [19]. Moreover, Geller et al. found in an analysis of seven mammography registries that despite recommendations by professional organizations, many women have not returned for mammography after treatment for breast cancer [20]. Taking into consideration that a majority affirmed the general necessity of surveillance for breast cancer in the conducted surveys, a quarter of the organized (25%) and a third of the non-organized (33%) patients responded contrary, that they prefer to neglect the disease. Moreover, about a third of the respondents stated that they feel confident and that the disease has been overcome (34%, e.g. 28% of the organized patients).

A major limitation of this investigation is due to the fact that only one third of the patients responded to the questionnaire. It is critical to discuss that probably those patients responded who are engaged in the topic of follow-up. The absence of a designated recall for non-responders therefore remains an open point of discussion. Furthermore, a majority of the patients who returned the questionnaire were organized members of a self-help group which potentially explains the extensive technical effort performed in those patients during follow-up. Despite these limitations, the present data reflect the ethical struggle of the physicians as well as the wishes of the afflicted patients and are therefore providing a solid basis for a new surveillance study.

## Conclusion

In summary one can conclude that women with a history of breast cancer have conflicting views on follow-up. Moreover clinicians obviously do not agree with the conclusions of older studies on which present guidelines are based. Lacking new evidence it is about time to initiate a new randomized surveillance study which investigates the efficacy of an intensified surveillance based on the improved possibilities of modern diagnostics and endocrine, immunotargeted, chemotherapeutical and interventional treatment options. Not only the physicians, but specifically also the women with breast cancer are prepared and determined to reanalyse this important field of oncological activity.

## Figures and Tables

**Table 1:** Question: Routine surveillance tests performed during follow-up (n = 801).

	**Self-help group (n = 476) [%]**	**Non-organized (n = 325) [%]**	**p [χ^2^-test]**
• **Clinical tests**			
Physical examination	88.2	85.5	ns (p = 0.26)
Routine history	97.9	92.9	p = 0.005
• **Laboratory tests**			
Laboratory tests	90.8	82.5	p = 0.005
Tumour marker tests	86.9	56.0	p < 0.0005
• **Imaging procedures**			
Chest x-ray	66.0	52.0	p < 0.0005
Ultrasound	92.2	85.5	p = 0.002
Bone scan	61.1	35.7	p < 0.0005
Computed tomography	35.7	29.5	ns (p = 0.07)
PET-CT scan	9.0	12.0	ns (p = 0.17)
Mammography	94.3	89.2	p = 0.008

ns: not significant
